# Controlling the Morphologies of Silver Aggregates by Laser-Induced Synthesis for Optimal SERS Detection

**DOI:** 10.3390/nano9111529

**Published:** 2019-10-27

**Authors:** Longkun Yang, Jingran Yang, Yuanyuan Li, Pan Li, Xiaojuan Chen, Zhipeng Li

**Affiliations:** Beijing Key Laboratory of Nano-Photonics and Nano-Structure (NPNS), Department of Physics, Capital Normal University, Beijing 100048, China

**Keywords:** silver aggregates, laser-induced synthesis, surface-enhanced Raman scattering, hot spots

## Abstract

Controlling the synthesis of metallic nanostructures for high quality surface-enhanced Raman scattering (SERS) materials has long been a central task of nanoscience and nanotechnology. In this work, silver aggregates with different surface morphologies were controllably synthesized on a glass–solution interface via a facile laser-induced reduction method. By correlating the surface morphologies with their SERS abilities, optimal parameters (laser power and irradiation time) for SERS aggregates synthesis were obtained. Importantly, the characteristics for largest near-field enhancement were identified, which are closely packed nanorice and flake structures with abundant surface roughness. These can generate numerous hot spots with huge enhancement in nanogaps and rough surface. These results provide an understanding of the correlation between morphologies and SERS performance, and could be helpful for developing optimal and applicable SERS materials.

## 1. Introduction

Surface-enhanced Raman scattering (SERS) is a powerful, nondestructive analytical tool owing to its high molecular specificity and sensitivity [1,2,3]. It has demonstrated promising applications in the fields of single-molecule spectroscopy [4,5], biochemical analysis [6,7,8,9,10], environmental monitoring [11,12,13,14], food safety [15,16], and even monitoring the reaction process at a molecular level [17,18,19,20]. The phenomenon of SERS is generally explained by a combination of electromagnetic [21,22,23,24] and chemical [25,26,27] enhancements. The former involves the enhancement of the electric field by the surface plasmons resonance of metallic nanoparticles. Especially, when two nanostructures are brought together, a giant local field can be generated in the gap or crevice due to the surface plasmons coupling, which is a hot spot for SERS detection [28,29,30,31,32]. The latter mainly originates from the charge transfer between the adsorbates and metal surface [25,26,27]. With respect to electromagnetic enhancement, a number of techniques have been developed to rationally design the SERS substrates with a large density of hot spots in order to improve the sensitivity and reproducibility of SERS measurements [33,34,35,36,37,38,39]. For instance, metallic nanostructures with various shapes, such as silver and gold spheres [40,41], cubes [42,43], polyhedrons [44,45], rods [46,47], and wires [48,49] have been chemically synthesized. When dropping the colloidal suspensions on an omniphobic or slippery substrate, SERS hot spots can be formed when the nanoparticles self-assemble during solvent evaporation [50,51]. On this slippery SERS platform, reproducibility can reach 25% for single-molecule SERS detection (~10^−13^ M) and can rapidly increase to >90% at higher detection concentrations (>10^−12^ M) [50]. On the other hand, it has been reported that silver aggregates with dense hot spots can directly grow on the interface of indium tin oxide (ITO) and reaction solution by a simple laser-induced photochemical reduction [52,53,54,55]. This laser-direct writing method provides a rapid, controllable, and low-cost way to synthesize SERS active materials. More importantly, this technique can integrate the SERS substrates directly into the microfluidic channel in a controlled fashion to create a lab-on-a-chip SERS system. It has the advantages of in situ preparation, automation, and real time detection, and avoids the unexpected contamination or oxidation degrading of SERS substrates, thus enables reproducible and sensitive SERS measurements [56,57,58]. With this silver aggregates-based SERS chip, the reproducibility of single-molecule SERS measurements can be raised up to ~50% [59]. We know the growth of silver aggregates on a glass–solution interface is highly dependent on laser power and irradiation time. Hence, it is critical to understand the correlation between the morphologies of aggregates and the corresponding SERS ability to optimize the performance of SERS materials synthesized by this laser-induced photochemical reduction method.

In this work, the laser-induced growths of silver aggregates on an ITO–solution interface were systematically investigated by tuning the power and irradiation time (532 nm laser). These structures can generate numerous hot spots at both the nanogaps and rough surface. By correlating the aggregates morphologies with their SERS abilities, the critical structure characteristics for large near-field enhancement were identified, which were closely packed nanorice and flake structures with abundant surface roughness. The understanding of the relation between morphology and SERS performance would be beneficial for developing optimal and applicable SERS materials. 

## 2. Experimental Section

### 2.1. In Situ Synthesis of Silver Aggregates 

Silver nitrate and sodium citrate dihydrate of analytical grade were bought from Sigma-Aldrich. Deionized water was used to prepare the solutions. The reactant mixture was obtained by mixing aqueous solutions of silver nitrate (0.1 mM) and sodium citrate (0.08 mM) in a 1:1 volume ratio. Then, a drip of reactant mixture was placed in a cell made up of a slide of ITO glass and a cover glass. A 532 nm continuous wave laser was focused on the ITO glass through an objective with 50× magnification (N.A. = 0.5). The power was tuned in the range of 0.4–0.9 mW by an attenuator. Laser irradiation time was set in the range of 30–180 s. The final products on ITO glass were rinsed for 5 min with deionized water to remove excess reactants. Using a scanning electron microscope (SEM, S-4800, 10 kV, Hitachi, Japan), the morphologies of silver aggregates synthesized under different power and irradiation time were characterized. With the help of coordinates on ITO glass, each characterized silver nanoaggregates could be specifically found again under an optical microscope. 

### 2.2. SERS Measurements

The SERS measurements were performed on an inVia Renishaw Raman Spectrometer at the excitation of a 532 nm laser (Renishaw, UK). A 50× magnification (N.A. = 0.5) objective was used. Here, the laser for Raman excitation was the same as the one used for photo reduction. The Raman excitation power was about 14 μW and integration time was 10 s, unless stated otherwise. Crystal violet (CV) with a concentration of 10^−7^ M in ethanol was chosen as the SERS analyte. The SERS sample was prepared by dropping 20 μL CV solution onto the ITO slide with silver aggregates. After it dried under ambient conditions, the area of the dried spot was about 1 cm^2^. For polarization measurements, the SERS spectra were repeatedly detected at the same position by changing the excitation polarization. To minimize the photobleaching-induced SERS decay [33], the integration time was set to 1 s. The intrinsic polarization dependence of the Raman instrument was calibrated by the Raman peak of silicon (111) surface.

## 3. Results and Discussion

**Figure 1:**
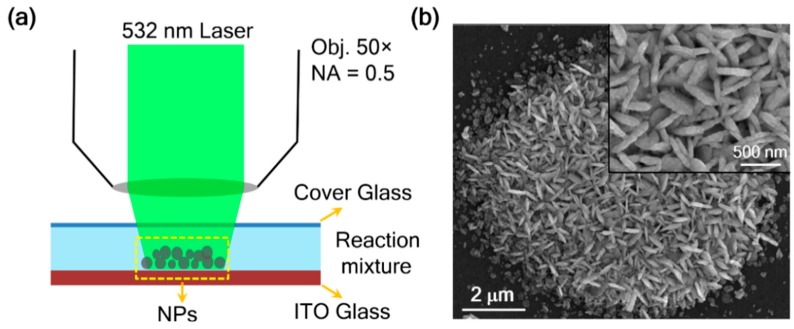
Laser-induced growth of silver aggregates. (**a**) Schematic of the experimental setup. (**b**) SEM image of a typical product fabricated by 60 s exposure at a laser power of 0.9 mW. Inset is a zoomed view of the silver aggregates.

Our experimental setup for the laser-induced synthesis of silver aggregates is schematically shown in Figure 1a (for details, see Experimental Section). A 532 nm continuous wave laser was focused onto the cell filled with reactant solution prepared by silver nitrate and sodium citrate. Then, the citrate reduced the silver ions to atoms at room temperature via a photooxidation mechanism, dissociating a hydrogen ion from the hydroxyl group on the citrate and converting it to acetone-1,3-dicarboxylate and carbon dioxide [60,61,62]. With the continuous increase of silver atoms, silver aggregate structures grew on the ITO–solution interface in a few seconds, and were observed under the microscope. The SEM image of a typical product synthesized by 60 s exposure at a laser power of 0.9 mW is shown in Figure 1b. From the SEM images, we found that the aggregate spot was about 10 μm in size and was made up of numerous nanorice and flake structures, with an average length of about 460 nm. These surface textures could form dense gaps or crevices and tips, which could generate a huge number of hot spots for SERS detection.

**Figure 2:**
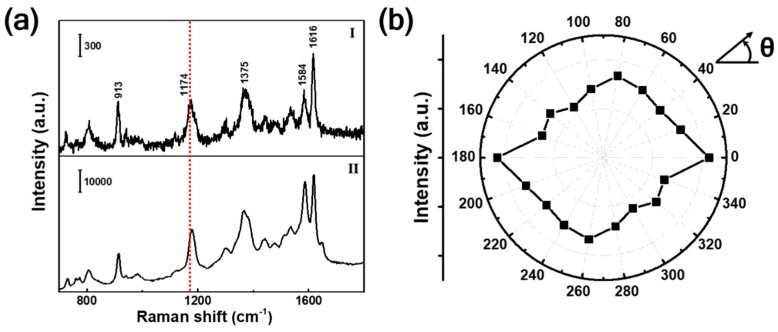
Surface-enhanced Raman scattering (SERS) measurements of the silver aggregates fabricated by 60 s exposure at a laser power of 0.9 mW. (**a**) Curve I: Raman spectrum of CV powder. Curve II: The SERS spectrum of CV adsorbed on the silver aggregates. (**b**) Polar plot of SERS intensity (1174 cm^−1^) under different excitation polarizations (θ).

The SERS performances of the prepared silver aggregates were then experimentally characterized by using 10^−7^ M CV as the probe. Curve I in Figure 2a shows the raw Raman spectrum obtained from CV powder. The Raman fingerprints at 913, 1174, 1375, 1584, and 1616 cm^−1^ were identified and were mainly from the vibrations of benzene ring [63]. The corresponding SERS spectrum of CV on the as-prepared silver aggregates is shown in curve II. By comparing to the spectrum from powder, we found that the Raman scattering intensity was greatly enhanced. According to the absorption spectrum of CV (Figure S1), resonant Raman scattering can be obtained under the excitation of 532 nm. Hence, the enhancement should come from the combined contributions of plasmonic effect and molecular resonance effect, which is surface-enhanced resonance Raman scattering. Generally, the enhancement factor (EF) can be evaluated by the following equation:

(1) $$\mathrm{EF}=\frac{I_{SERS}}{I_{Bulk}}\times \frac{N_{Bulk}}{N_{SERS}}\;,$$

where *I_SERS_* and *I_Bulk_* are the intensity of a Raman mode with and without surface enhancement, respectively, and *N_SERS_* and *N_Bulk_* refer to the corresponding number of CV molecules [64,65,66]. By choosing the CV band at 1174 cm^−1^ as a reference, the EF was estimated to be 2.0 × 10^7^ (see supporting information for details). Considering that the bulk Raman was also excited by 532 nm laser, the molecular resonance effect would be offset to some extent in EF evaluation. Hence, the EF calculated by Equation 1 can be attributed to electromagnetic enhancement of silver aggregates. Here, we should emphasize that the EF is an average value over the whole surface of silver aggregates. The enhancements on the tips of nanorices and flakes or inside the gaps of nanoaggregates would be much larger. As is known, the electromagnetic enhancement of metallic nanostructures is highly dependent on excitation polarization [67,68]. Hence, the polarization-dependent SERS of these silver aggregates were investigated. The normalized SERS intensity of CV under different incident polarizations are shown in the polar plot (Figure 2b). Unlike the highly polarization-dependent single-nanogap system [68], the aggregates structure was insensitive to the excitation polarization with the SERS intensity fluctuation at orthogonal polarizations less than 20%. This is to be expected because the as-prepared silver aggregates were made up of dense nanogaps or crevices with random sizes and orientations (Figure 1b).

**Figure 3:**
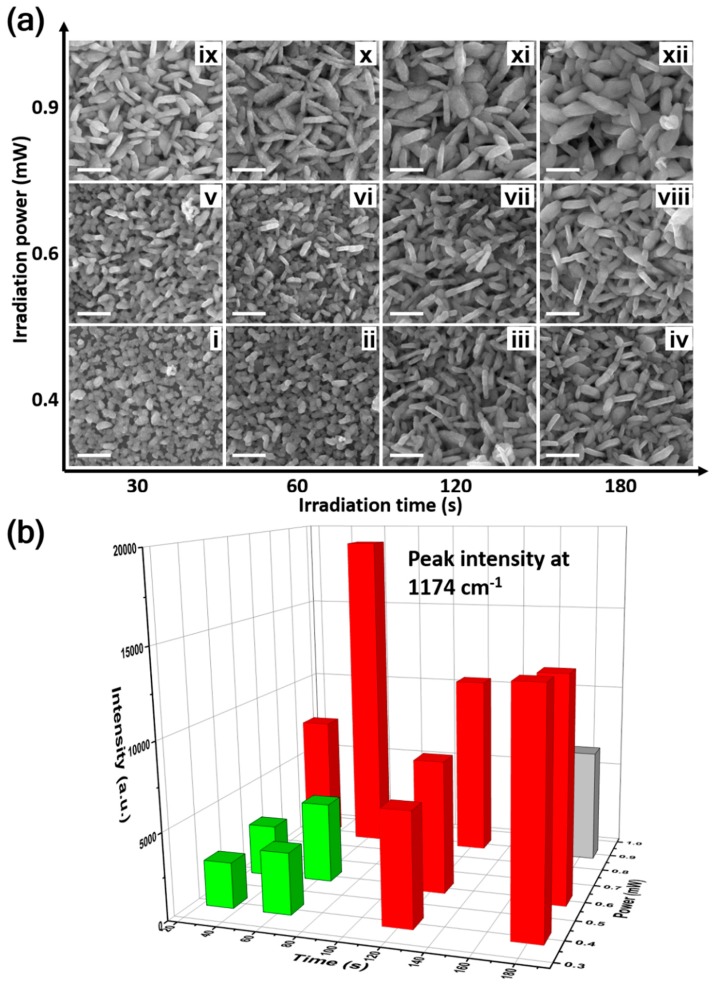
Controlling synthesis of silver aggregates and their SERS activities. (**a**) SEM images of silver aggregates grown under different irradiation power (0.4–0.9 mW) and exposure time (30–180 s). The scale bars are all 500 nm. (**b**) The corresponding SERS intensity from the silver aggregates are shown in (**a**).

To seek the optimal synthesis parameters, SERS aggregates were created under different laser power (0.4, 0.6, and 0.9 mW) and irradiation time (30, 60, 120, and 180 s). The morphologies of the prepared aggregates are summarized in Figure 3a. To clarify the influence of the laser power and irradiation time, we first analyzed the morphological changes as the increase of irradiation time at certain laser power. Such as the aggregates (i–iv), under the parameters: power = 0.4 mW, time = 30 s (i), a layer of silver nanoparticles with an average diameter of ~100 nm first formed on the ITO substrate. Then, for a longer irradiation time of ~60 s (ii), these nanoparticles became denser, and some nanorice structures began to emerge. Further increasing the irradiation time to 120 and 180 s (iii and iv) resulted in the nanorices growing larger and denser, with the length reaching up to ~400 nm. We then focused on the influence of irradiation power. The morphological changes that occurred as the laser power increased at certain irradiation times are shown by column. Along with aggregates i, v and ix, we also observed morphological changes from nanoparticles to nanorices. Based on these morphological evolutions, we deduced that the growth of silver aggregates was tuned by the photon dose through the combination effect of photoinduced growth and coalescence [53,69,70]: ① The silver nuclei were first formed in solution and then grown into nanoparticles through Ostwald ripening. ② As the nanoparticles grew, the adjacent particles began to coalesce with each other and formed linear polycrystalline structures called nanorices. Some concaves between the connected particles can still be observed from the SEM images of aggregates ii, iii, v and vi (see the magnified images in Figure S2 for details). ③ The nanorices grew via the atoms and/or nuclei addition. Finally, some nanoflake structures were formed. Here, we should note that the growth rate of the nanostructures can be tuned by the concentration of the reducing agent (citrate) [53]. As shown in Figure S3, we monitored the growth of silver aggregates under different citrate concentrations (0.01, 0.08, and 0.64 mM). The dark-field scattering images show that aggregates grew slowly under lower citrate concentrations. Under relatively high citrate concentrations, dense aggregates can form quickly in tens of seconds. In our experiments (data in Figure 3), a moderate citrate concentration (0.08 mM) was adopted to provide better controllability by the laser power and irradiation time.

Then, the SERS performance of these synthesized silver aggregates was investigated using 10^−7^ M CV as a probe. The corresponding SERS spectra are shown in Figure S4. Figure 3b presents the statistics of the peak intensity at 1174 cm^−1^. Depending on the SERS intensity, the silver aggregates can be separated into two groups. For the first group (i, ii, v, vi), the enhancement was relatively low, with SERS intensity in the range of 2600–4600, where the aggregates were dominated by nanoparticles. Interestingly, the second group with compact nanorices aggregates (iii, iv, vii, viii, ix, x, xi) exhibited prominent SERS signals, among which the largest SERS intensity could reach up to 19,000 (x). From the SEM images in Figure 3a, we noticed that the aggregates x was composed of closely packed nanorice and flake structures with abundant surface roughness. This can be understood by the fact that the roughened nanorices and flakes aggregates can enhance the local field in two ways. One is the smaller nanogaps/crevices with higher near-field enhancement. On average, the compact nanostructures in group two (such as the aggregates x) generate smaller gaps than the sparsely small nanoparticles in group one (such as aggregates i), as shown in Figure S5. The other is from the abundant rough structures on the nanorices and flakes surface. The contribution of surface roughness to near-field enhancement on a mesostructure has also been confirmed by previous experiments and simulations [71,72,73]. Numerical simulations were also performed to help visualize the enhancement contributions from gaps and rough surface. As shown in Figure S6, the two coupled nanorices generated obvious near-field enhancement at both the gap and rough surface positions. While, the SERS intensity decreased in the case of aggregates xii, though the laser irradiation time (180 s) was longer than that of aggregate × (60 s). This could be caused by the disappearance of surface roughness during nanostructures overgrowth. The zoomed in images of aggregates x and xii are compared in Figure S5. Additionally, the overgrowth of nanostructures can also quench some nanogap enhancement due to the direct contact between the nanostructures. 

## 4. Conclusions

In summary, a highly active silver aggregates SERS material was directly synthesized on the ITO–solution interface via a facile in situ photochemical reduction method. The morphologies of these aggregates were effectively controlled by laser power and irradiation time. By correlating the morphologies with their SERS signals, the best SERS aggregates were obtained under the synthesis parameters: power = 0.9 mW, time = 60 s. The average SERS EF was as large as 2.0 × 10^7^. Importantly, the morphology features of optimal SERS aggregates were identified. Aggregates composed of packed nanorices and flakes with abundant surface roughness would possess better SERS ability. An understanding of the relation between morphology and SERS performance would be beneficial for controlled synthesis of optimal SERS materials with a high density of hot spots, and the development of practical SERS techniques.

## Supplementary Materials

The following are available online at https://www.mdpi.com/2079-4991/9/11/1529/s1. Contents: SERS enhancement factor estimation. Figure S1. The absorption spectrum of 10^−4^ M CV in water; Figure S2. The morphological evolutions of silver aggregates; Figure S3. Monitoring the silver aggregates growths under different citrate concentrations; Figure S4. The SERS spectra measured on the silver aggregates synthesized under different parameters; Figure S5. The magnified images of aggregates i, x and xii; Figure S6. The numerical simulations of the local field distributions around the two coupled roughened nanorices.
