# Supplementary material for: Controlling the Morphologies of Silver Aggregates by Laser-Induced Synthesis for Optimal SERS Detection
Source: Nanomaterials (Basel). 2019 Oct 27;9(11):1529. doi: 10.3390/nano9111529 (PMC6915404; doi:10.3390/nano9111529)
Supplement: Supplementary file 1 [file nanomaterials-09-01529-s001.pdf]

Supplementary materials

# Controlling the morphologies of silver aggregates by laser-induced synthesis for optimal SERS detection

**Longkun Yang†, Jingran Yang†, Yuanyuan Li, Pan Li, Xiaojuan Chen, Zhipeng Li***

Beijing Key Laboratory of Nano-Photonics and Nano-Structure (NPNS), Department of Physics, Capital Normal University, Beijing 100048, PR China

* Correspondence: zpli@cnu.edu.cn

† These authors contributed equally to this work.

**SERS enhancement factor estimation**

Generally, the SERS EF can be evaluated by the equation as follow:

$$\mathrm{EF} = \frac{I_{SERS}}{I_{Bulk}} \times \frac{N_{Bulk}}{N_{SERS}} \quad (1),$$

where $I_{SERS}$ and $I_{Bulk}$ are the intensity of a Raman mode with and without surface enhancement, respectively, $N_{SERS}$ and $N_{Bulk}$ refer to the corresponding number of CV molecules. Here, the $N_{Bulk}$ is calculated by $N_A(\rho V_{Laser}/M)$, where $N_A$ is the Avogadro number, ρ and M are the density (1.19 $g/cm^{-3}$) and molecular weight (408 g/mol) of CV, $V_{Laser}$ is the focal volume of the laser illumination (in our experiments, the diameter of laser spot is about 3 μm and the illumination depth is estimated to be 3 μm). On the other hand, for the $N_{SERS}$ estimation, we assume that the CV molecules are distributed evenly across the dried spot. So the $N_{SERS}$ can be calculated as follow:

$$N_{SERS} = N_A \times C_{CV} \times V_{CV} \times \frac{S_{Laser}}{S_{Dried-spot}} \quad (2),$$

in which $C_{CV}$ and $V_{CV}$ are the concentration and volume of CV solution used for SERS sample preparation, and $S_{Laser}$ and $S_{Dried\text{-}spot}$ are the area of laser spot and dried-spot of CV solution. According to the SERS and Raman measurements in Figure 2a, the enhancement factor can be estimated to be as large as $2.0\times10^7$.

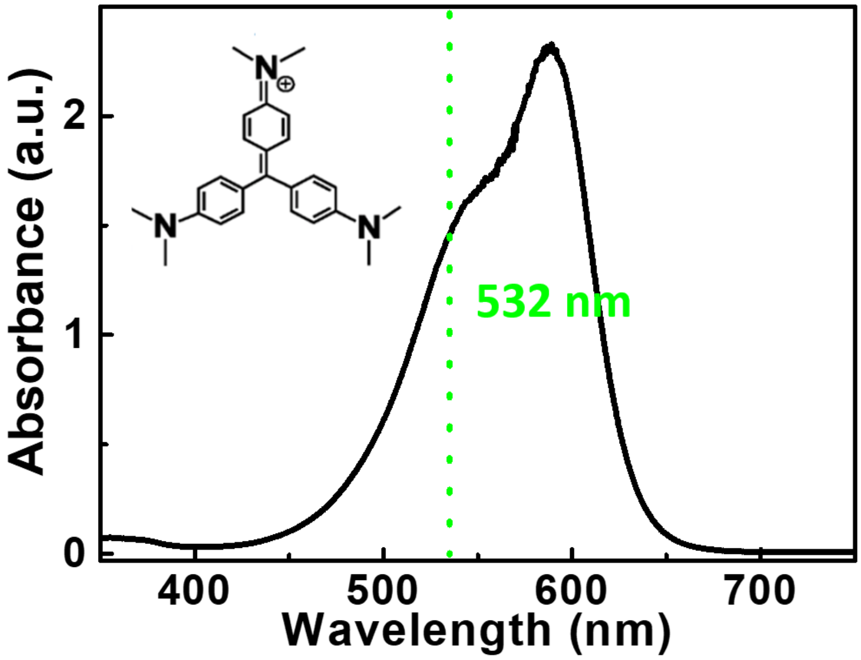


**Figure S1.** The absorption spectrum of $10^{-4}$ M crystal violet in water. The dash line indicates the position of Raman excitation.

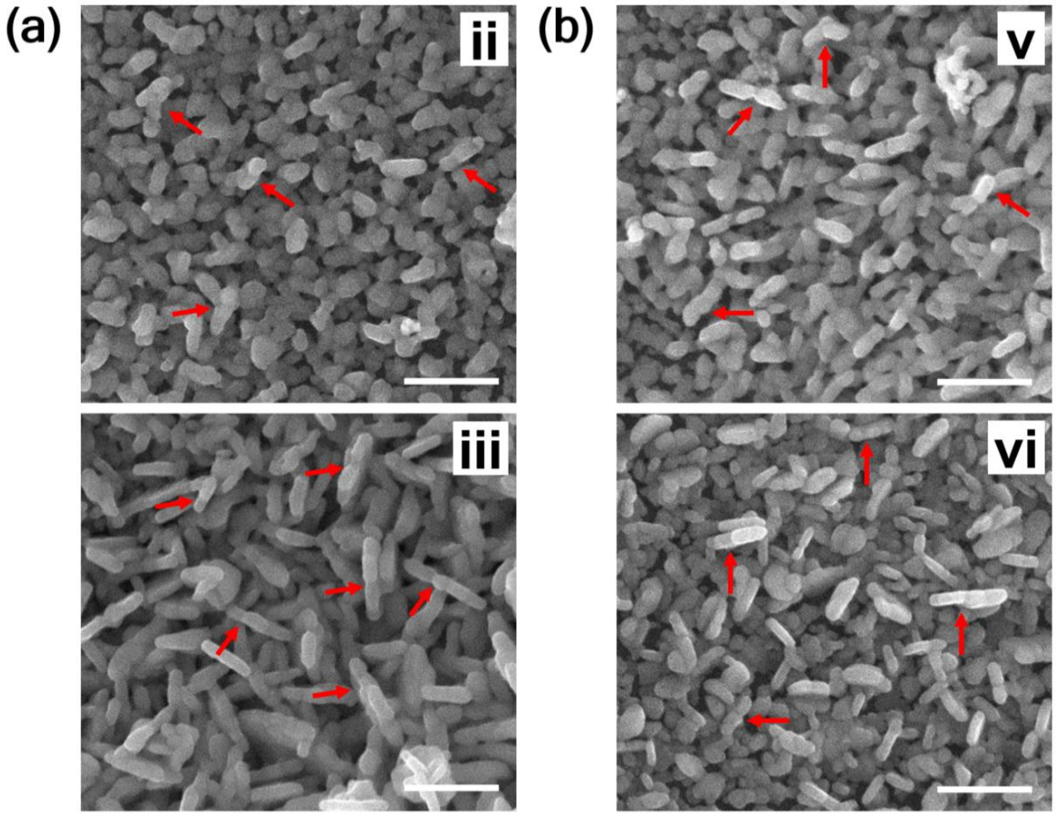


**Figure S2.** The morphological evolutions of silver aggregates. (**a**) The laser power is 0.4 mW. The irradiation times for ii and iii are 60 and 120 s, respectively. (**b**) The laser power is 0.6 mW. The irradiation times for v and vi are 30 and 60 s, respectively. The red arrows indicate the tracks of the nanoparticles coalescence. The scale bars are all 500 nm.

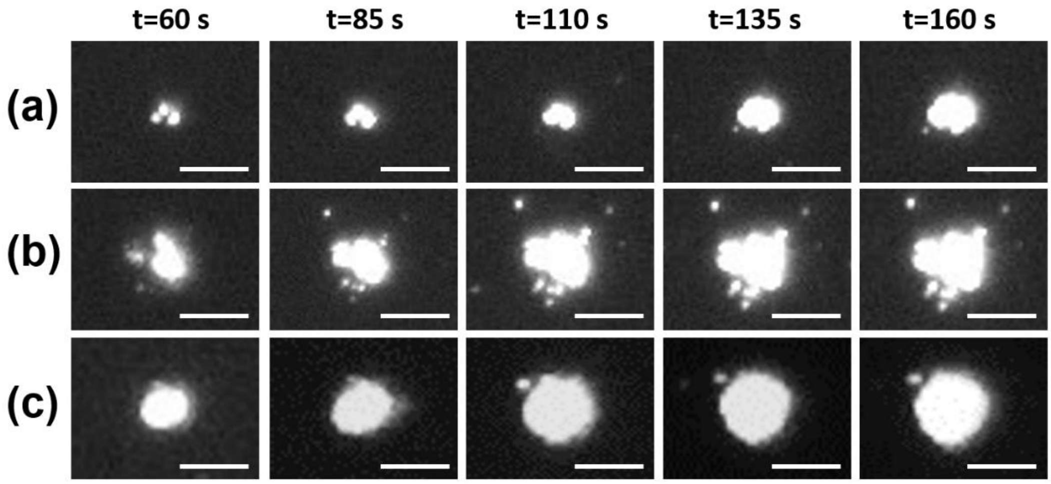


**Figure S3.** Monitoring the silver aggregates growths under different citrate concentrations: (**a**) 0.01 mM; (**b**) 0.08 mM; (**c**) 0.64 mM. The concentrations of silver nitrate were all 0.1 mM. The laser power was 0.9 mW. The dark-field scattering images were captured at different irradiation times: t= 60 s, 85 s, 110 s, 135 s and 160 s, respectively. The scale bars are all 10 μm.

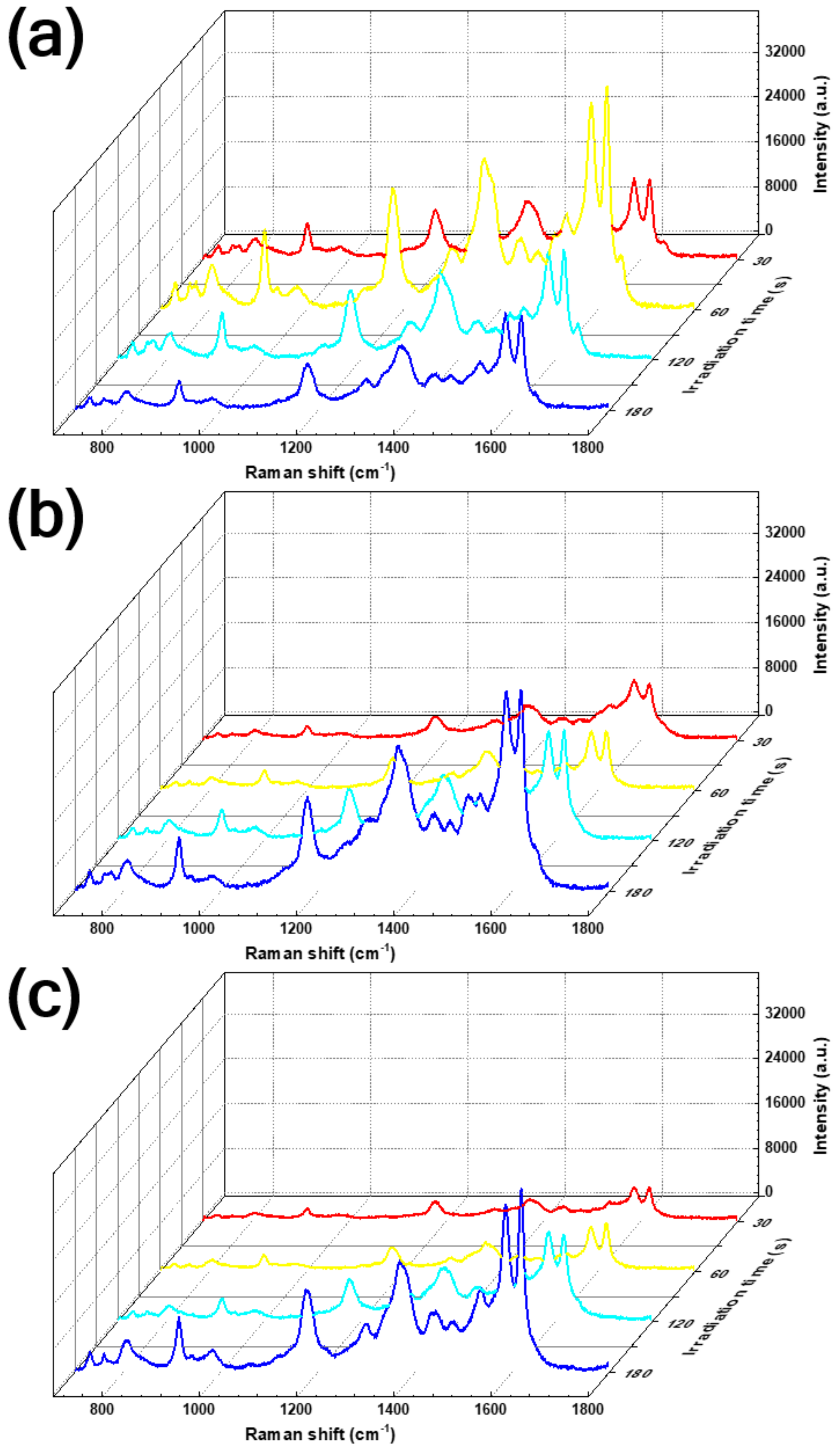


**Figure S4.** The SERS spectra measured on the silver aggregates synthesized under different laser power and irradiation time. The laser power is 0.9 mW (**a**), 0.6 mW (**b**) and 0.4 mW (**c**).

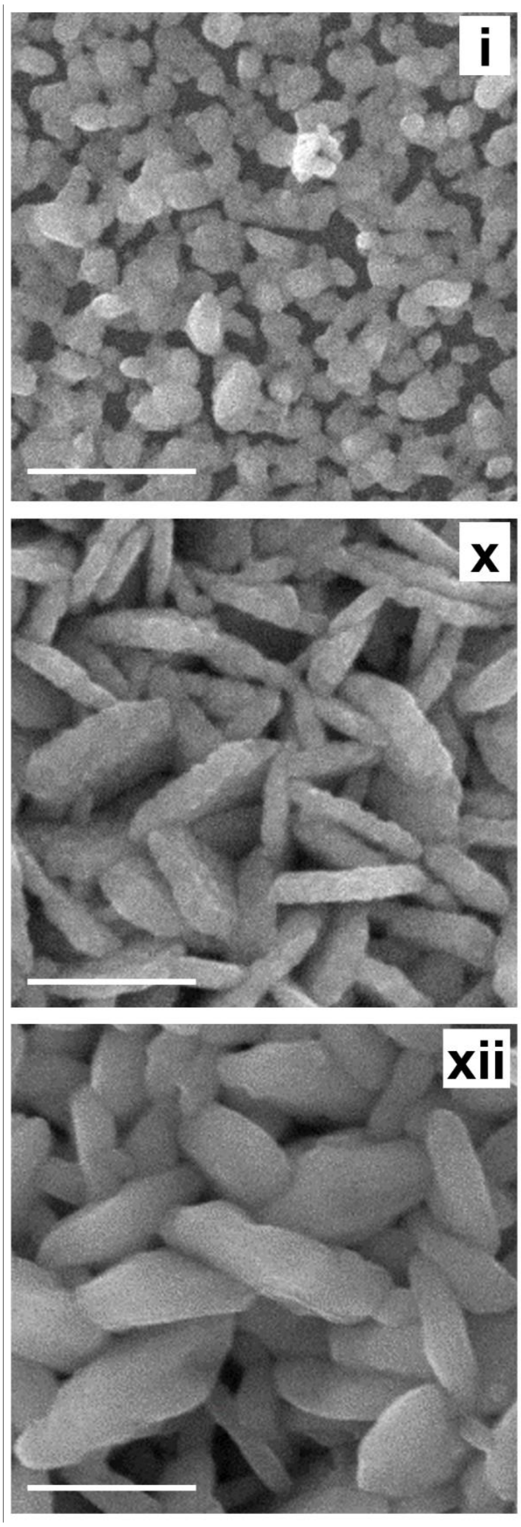


**Figure S5.** The magnified images of aggregates i, x and xii. The scale bars are all 500 nm.

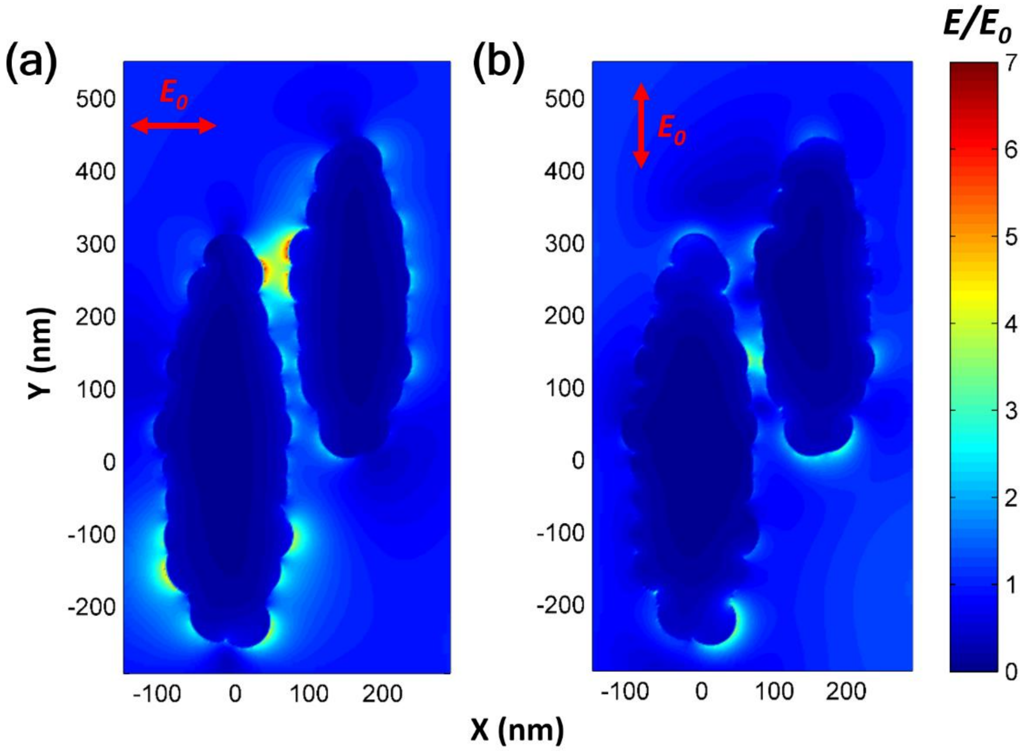


**Figure S6.** The finite-difference time-domain simulations of the local field distributions ($E/E_0$) around the two coupled roughened nanorices under perpendicular (**a**) and parallel (**b**) excitation polarizations. The lengths of the nanorices are 460 nm and 350 nm, respectively. The Johnson-Christy dielectric data for silver were used, and a vacuum with real refractive index 1 was selected for the background medium. Here, to clearly present the near-field enhancement in gaps and rough surface, weakly coupled nanorices with separation ~20 nm are simulated. In experiments, the averaged gap can be smaller (several nanometer), which would generate higher enhancement.
